# Development of a speed breeding protocol with flowering gene investigation in pepper (*Capsicum annuum*)

**DOI:** 10.3389/fpls.2023.1151765

**Published:** 2023-09-18

**Authors:** Hayoung Choi, Seungki Back, Geon Woo Kim, Kyeongseok Lee, Jelli Venkatesh, Hyo Beom Lee, Jin-Kyung Kwon, Byoung-Cheorl Kang

**Affiliations:** Department of Agriculture, Forestry and Bioresources, Research Institute of Agriculture and Life Sciences, Plant Genomics and Breeding Institute, College of Agriculture and Life Sciences, Seoul National University, Seoul, Republic of Korea

**Keywords:** pepper, speed breeding, photoperiod, far-red light, genome-wide association study, gene expression analysis

## Abstract

Pepper (*Capsicum* spp.) is a vegetable and spice crop in the Solanaceae family with many nutritional benefits for human health. During several decades, horticultural traits, including disease resistance, yield, and fruit quality, have been improved through conventional breeding methods. Nevertheless, cultivar development is a time-consuming process because of the long generation time of pepper. Recently, speed breeding has been introduced as a solution for shorting the breeding cycle in long-day or day-neutral field crops, but there have been only a few studies on speed breeding in vegetable crops. In this study, a speed breeding protocol for pepper was developed by controlling the photoperiod and light quality. Under the condition of a low red (R) to far-red (FR) ratio of 0.3 with an extended photoperiod (Epp) of 20 h (95 ± 0 DAT), the time to first harvest was shortened by 75 days after transplant (DAT) compared to that of the control treatment (170 ± 2 DAT), suggesting that Epp with FR light is an essential factor for flowering in pepper. In addition, we established the speed breeding system in a greenhouse with a 20 h photoperiod and a 3.8 R:FR ratio and promoted the breeding cycle of C. *annuum* for 110 days from seed to seed. To explain the accelerated flowering response to the Epp and supplemented FR light, genome-wide association study (GWAS) and gene expression analysis were performed. As a result of the GWAS, we identified a new flowering gene locus for pepper and suggested four candidate genes for flowering (*APETALA2* (*AP2*), *WUSCHEL*-*RELATED HOMEOBOX4* (*WOX4*), *FLOWERING LOCUS T* (*FT*), and *GIGANTEA* (*GI*)). Through expression analysis with the candidate genes, it appeared that Epp and FR induced flowering by up-regulating the flowering-promoting gene *GI* and down-regulating *FT*. The results demonstrate the effect of a combination of Epp and FR light by genetic analysis of flowering gene expression. This is the first study that verifies gene expression patterns associated with the flowering responses of pepper in a speed breeding system. Overall, this study demonstrates that speed breeding can shorten the breeding cycle and accelerate genetic research in pepper through reduced generation time.

## 1 Introduction

Pepper is a great source of vitamin C and β-carotene, and the only plant genus that synthesizes capsaicinoids (Srivastava and Mangal, 2019). Pepper capsaicinoids are used in food ingredients due to their unique taste, and also have physiological and pharmacological health benefits including anticancer, anti-inflammatory, and anti-obesity properties (Aza-González et al., 2011). Owing to these nutritional benefits, producing high-quality cultivars is one of the major goals of pepper breeding.

The long generation time of pepper is a bottleneck in breeding programs; it takes 7-8 years to develop a cultivar. To mitigate this issue, there have been various technologies to shorten the generation time. The most well-known approach is shuttle breeding, which uses two distinct fields with different climates (Ortiz et al., 2007). Shuttle breeding enables only two generations per year by growing breeding populations at two locations where plants can produce seeds (Ortiz et al., 2007). For a successful shuttle breeding approach, two fields with different environmental requirements are needed for testing and generation advancement, which can be laborious and expensive. Doubled haploid (DH) technology can generate homozygous lines rapidly by bypassing the process of inbreeding (Gerald et al., 2013). However, each step of DH is strongly genotype-dependent, and this method is laborious, expensive, and requires high skills to obtain a successful product (Gerald et al., 2013). Genome-editing technologies can also generate desired plants by directly editing target genes (Araki and Ishii, 2015). However, this approach is difficult to applying to certain species and requires skilled technicians (Araki and Ishii, 2015). To overcome these issues, Watson et al. (2018) proposed a simple but effective technology, ‘speed breeding’.

The response of crops to continuous light conditions has been the subject of scientific investigation for many years. In the 1980s, NASA conducted an experiment in which crops, including wheat, soybean, lettuce, and potato, were grown under constant light conditions. It was observed that the total biomass of these crops strongly depended on the amount of light provided to the plants (Wheeler et al., 1996). O'Connell (2007) focused specifically on exploring how wheat plants respond to continuous light and investigated the potential implications of continuous light on wheat growth and development. O'Connor et al. (2013) described a speed breeding technique that utilized controlled environment conditions, continuous light, and a single seed descent breeding strategy in a greenhouse to reduce the generation time of full-season maturity peanut cultivars from 145 to 89 days, potentially accelerating the development of new varieties. Inspired by previous works, Watson et al. (2018) coined the term ‘speed breeding’ and developed protocols for shortening the generation time of long-day field crops by extending the photoperiod. They achieved up to 6 generations per year for wheat (*Triticum aestivum*), barley (*Hordeum vulgare*), chickpea (*Cicer arietinum*), and pea (*Pisum sativum*) compared to the 2 to 3 generations of traditionally grown crops. Although speed breeding is a powerful alternative for reducing generation time in long-day plants, this method has rarely been applied to short-day plants. Recently, Jähne et al. (2020) demonstrated a protocol for short-day crops soybean (*Glycine max*), rice (*Oryza sativa*), and amaranth (*Amaranthus* spp.), adjusting the photoperiod to 10 h and adjusting the light intensity and quality. Far-red (FR) light allowed flowering time reduction in some amaranth and rice genotypes, but there was no impact on soybeans. Therefore, crop-specific LED lighting schemes will need to be developed (Jähne et al., 2020). Previous studies on speed breeding primarily focused on field crops, but research for vegetable crops is scarce. Liu et al. (2022) developed a speed breeding scheme for hot pepper through light environment modification. They investigated the growth and development of hot pepper varieties (‘Xiangyan 55’ and ‘Xiangla 712’) under various light intensities (240, 300, 360, and 420 PPFD), photoperiods (14 h, 16 h, 18 h, and 20 h), and R:FR ratios (2.1, 2.7, 3.8, and 6.3) (Liu et al., 2022). They reported that light intensities of 300 and 420 PPFD, a photoperiod of 20h, and an R:FR ratio of 3.8 were effective in shortening the flowering time for hot peppers (Liu et al., 2022). However, they did not confirm the effect of combining all the conditions of light intensity, photoperiod, and supplementary FR. Since the plant’s response to the speed breeding conditions in growth chambers or plant factories can be different from in actual greenhouse conditions, further study for applying comprehensive speed breeding in the greenhouse needs to be done. Moreover, the genetic and molecular basis of speed breeding has not been fully elucidated.

Flowering time is controlled by the interaction of endogenous and exogenous signals such as photoperiod, temperature, and plant hormones (Srikanth and Schmid, 2011). Depending on the environmental signals, the pattern of flowering gene expression is diverse (Cho et al., 2017; Ding et al., 2020). Previous studies on Arabidopsis, a model plant, have revealed many flowering-associated genes and flowering gene expression patterns according to environmental controls (Mouradov et al., 2002). In contrast, there are few studies on the expression patterns of flowering genes in pepper under environmental change (extending day length, adding FR, etc.). Moreover, although flowering genes are conserved between species, they are likely to have very different functions in each species. For example, *SHORT VEGETATIVE PHASE* (*SVP*) acts as a major flowering repressor together with *FLOWERING LOCUS C* (*FLC*) in Arabidopsis (Li et al., 2008), while its homolog, *CaJOINTLESS*, acts as a major flowering activator in pepper (Cohen et al., 2012). Therefore, a genetic study is required for each particular species. Furthermore, it is necessary to analyze the changes in flowering gene expression in a controlled environment specific to each crop speed breeding system. This genetic analysis would identify the cause of accelerated flowering time under speed breeding.

In this paper, we established a speed breeding system suitable for pepper by shortening the generation time of pepper *via* extending the day length to 20 h, controlling light quality by supplementing FR, and adjusting the R:FR ratio. We then demonstrated the effectiveness of this protocol on a genetic and molecular basis. Moreover, we applied the speed breeding system in the greenhouse and established a speed breeding protocol for *C*. *annuum*, enabling us to shorten the breeding pipeline in less than three years. To identify the genetic loci associated with flowering time, we performed a genome-wide association study (GWAS) and investigated the expression levels of candidate genes for comparative analysis of the flowering gene expression patterns between the speed breeding system and the normal environmental growing conditions. It is expected that this study will have a significant impact on breeding and genetic research by shortening the breeding cycle, and the GWAS-identified candidate genes for loci associated with shortened flowering time provide valuable information about the genetic control of flowering time in pepper.

## 2 Materials and methods

### 2.1 Plant materials

For setting the speed breeding system for pepper, *Capsicum annuum* ‘MicroPep Red’ (‘MR’) from germplasm of the Horticultural Crops Breeding and Genetics Laboratory (Seoul National University, Republic of Korea) was used as the plant material of this study. ‘MR’ has a rapid generation time and short plant height compared to other cultivars.

For GWAS, 220 C. *annuum* accessions were used (Lee et al., 2020). The 220 accessions comprising the GWAS population were grown in a greenhouse at the RDA-GenBank (Jeonju, Republic of Korea) in 2015, 2016, and 2017. Over three years, six plants per accession were randomly planted, and three plants per accession were evaluated for flowering time (Lee et al., 2020).

### 2.2 Extending the photoperiod experiment

For each treatment, a multi-room chamber (Hanbaek, Bucheon, Republic of Korea) was programmed to have a day and night cycle of 12/12 h, 16/8 h, 20/4 h, and 24/0 h. In all the treatments, the temperature was set at 26°C during the day and 20°C during the dark (**Table 1**). The light source was a fluorescent lamp, and the light intensity was adjusted to 66.2 µmol·m^-2^·s^-1^ at bench height (**Table 1**). The seeds were sown in a 72-hole seedling tray, and the seedlings were grown for 20 days in a walk-in chamber under a 16/8-hour day/night cycle and temperature conditions of 25/20°C. Subsequently, individual seedlings were transplanted into pots with a diameter of 9.2 cm and a height of 8.8 cm. The pots were filled with approximately 90% of a commercial substrate called ‘Barokuh’ (SeoulBio, Eumseong, Republic of Korea). The composition and ratio of the ‘Barokuh’ substrate were as follows: zeolite 4%, perlite 7%, pumice 6%, coco peat 68%, peat moss 14.73%, fertilizer 0.201% (containing N, P, K, Ca, Mg, Fe, Cu, Zn, B, and other elements), wetting agent 0.064%, and pH adjuster 0.005%. The physical properties of the substrate included a moisture content range of 40-60%, an air-filled porosity range of 30-50%, and a bulk density of 0.15-0.25 Mg/m^3^. The chemical properties of the substrate were characterized by a pH range of 5.5-7.0 and an electrical conductivity (EC) of 0.65 ds/m. Throughout the cultivation period, no additional fertilizers were applied. Each pot was watered once daily in the morning using approximately 150-200 ml of tap water. Eight plants of ‘MR’ at two leaves (at least 1cm) were transplanted for testing the effect of extended photoperiod on flowering time and phenotyped for number of days to transition (floral meristem appearance) and flowering from days after transplanting (DAT).

**Table 1:** Growth conditions for extending the photoperiod, far-red (FR) light intensity, pepper-specific speed breeding system, and application of a speed breeding system in a greenhouse (GH).

Treatment	Photoperiod (h) (day/night)	Temperature (°C) (day/night)	Light source	Light intensity (µmol·m^-2^·s^-1^)			R:FR ratio
				White (W: 400- 700 nm)	Red (R: 600- 700 nm)	Far-red (FR: 700- 780 nm)	
12/12 h	12/12	26/20	Fluorescent lamp	66.2	15.3	2.7	5.7
16/8 h	16/8	26/20	Fluorescent lamp	66.2	15.3	2.7	5.7
20/4 h	20/4	26/20	Fluorescent lamp	66.2	15.3	2.7	5.7
24/0 h	24/0	26/20	Fluorescent lamp	66.2	15.3	2.7	5.7
W45	20/4	27/24	Warm-white LED	45.0	22.2	3.0	7.3
W45+LFR	20/4	27/24	Warm-white LED +FR LED	45.2	22.3	18.8	1.2
W45+MFR	20/4	27/24	Warm-white LED +FR LED	45.3	23.0	38.7	0.6
W45+HFR	20/4	27/24	Warm-white LED +FR LED	45.2	23.3	78.2	0.3
Ctl	12/12	26/20	Fluorescent lamp	66.2	15.3	2.7	5.7
Ctl+FR	12/12	26/20	Fluorescent lamp +FR LED	66.2	17.0	56.5	0.3
Epp	20/4	26/20	Fluorescent lamp	66.2	15.4	2.7	5.8
Epp+FR	20/4	26/20	Fluorescent lamp +FR LED	66.2	17.0	57.1	0.3
Normal GH	Natural light	Suwon farm condition	Natural light	152.8	55.1	40.3	1.4
Epp GH	20/4	Suwon farm condition	Natural light +LED (400-700 nm)	319.7	151.3	31.8	4.8
Epp+FR GH	20/4	Suwon farm condition	Natural light +LED (400-772 nm)	450.7	283.2	74.7	3.8

### 2.3 FR light intensity experiment

The FR light intensity experiment was conducted in the walk-in chamber at Seoul National University Farm (Suwon, Republic of Korea). This walk-in chamber was programmed to run a 20 h photoperiod and 4 h dark period with a temperature of 27°C during the day and 24°C during the night (**Table 1**). The relative humidity was set to 70%. Warm-white (W) and FR LEDs were used to test the effect of FR light on accelerating the transition from the vegetative to the reproductive stage. Light intensity (400-700nm) was set at 45 µmol·m^-2^·s^-1^ (W LEDs; W45) in all four treatments: W45 treatment (non-FR), W45+LFR (low FR), W45+MFR (medium FR), and W45+HFR (high FR) (**Table 1**). The light intensity of FR light in LFR, MFR, and HFR was 18.8, 38.7, and 78.2 µmol·m^-2^·s^-1^ each (**Table 1** and **Supplementary Figure 1**). The ‘MR’ seeds were directly sown in the 72 hole seedling tray and grown in each treatment of the walk-in chamber without undergoing a separate seedling growth process. The basic cultivation conditions, such as pot size, amount of media, composition of the media, and water management, remained consistent with those described in the “Extending the photoperiod experiment” section. Ten plants of ‘MR’ were grown in each treatment and plant height (distance from the shoot apical meristem to the soil) and time to floral meristem were recorded.

### 2.4 Pepper-specific speed breeding system

Plants were grown in a multi-room chamber (Hanbaek, Bucheon, Republic of Korea). For all the treatments: Control (Ctl), Control+Far-red light (Ctl+FR), Extended photoperiod (Epp), and Extended photoperiod+Far-red light (Epp+FR), each chamber room was programmed at 26°C during the day and 20°C at night (**Table 1**). In Ctl and Ctl+FR treatments, each chamber room was set to have a 12 h photoperiod and 12 h dark period (**Table 1**). In the case of the Epp and Epp+FR treatments, the day and night lengths were set to 20 h and 4 h (**Table 1**). In Ctl+FR and Epp+FR treatments, FR light was supplemented with R:FR=0.3 (**Table 1** and **Supplementary Figure 2**). The light intensity of the fluorescent lamp for all the treatments was adjusted to 66.2 µmol·m^-2^·s^-1^ (**Table 1**). The seeds were sown in the 72 hole seedling tray, and the seedlings were cultivated for 20 days in a walk-in chamber with a day/night cycle of 16/8 hours and temperature settings of 25/20°C. The basic cultivation conditions, such as pot size, amount of media, composition of the media, and water management, remained consistent with those described in the “Extending the photoperiod experiment” section. In each experimental treatment, a total of 10 ‘MR’ plants at the 1-2 leaf stage (with a minimum length of 1 cm) were transplanted. Various parameters were recorded, including the duration of the transition phase, the timing of the first flower appearance, the onset of first fruit production, the ripening of the fruits, the number of fruits produced, and the distance from the shoot apical meristem to the soil surface. The observed data pertaining to these parameters have been organized and presented in **Tables 2**, **3**. Specifically, the parameter labeled as ‘Transition’ denotes the critical moment when the floral meristem becomes visible, signaling the emergence of the first flower bud. ‘First flower’ represents the precise point in time when the initial flower blossoms on the plant. ‘First fruit’ characterizes the developmental stage when the plant generates its first fruit. ‘First ripening’ captures the significant stage at which the first fruit reaches full maturity, acquiring a vibrant red coloration referred to as the mature red stage.

**Table 2:** Development of *Capsicum annuum* ‘MicroPep Red’ (‘MR’) under the treatments of extending the photoperiod experiment, far-red (FR) light intensity experiment, pepper-specific speed breeding system, and application of speed breeding in greenhouse (GH).

Experiment	Treatment	Days after transplant to **			
		Transition	First flower	First fruit	First ripening
Extending the photoperiod experiment	12/12 h	28.9±2.5 ^a^	43.6±3.7 ^a^	–	–
	16/8 h	28.9±2.5 ^a^	43.1±4.3 ^a^	–	–
	20/4 h	28±0 ^a^	37.5±2.1 ^b^	–	–
	24/0 h	23.6±3.6 ^b^	36.5±2.1 ^b^	–	–
FR light intensity experiment (day/night: 20/4 h)	W45	25.9±3.4 ^a^	–	–	–
	W45+LFR	23.9±4.4 ^ab^	–	–	–
	W45+MFR	21.7±4.3 ^bc^	–	–	–
	W45+HFR	18.6±0.5 ^c^	–	–	–
Pepper-specific speed breeding system (day/night - Ctl: 12/12 h - Epp: 20/4 h)	Ctl	36±2 ^a^	56±2 ^a^	113±5 ^a^	170±2 ^a^
	Ctl+FR	29±5 ^b^	50±6 ^b^	106±8 ^a^	163±2 ^b^
	Epp	24±5 ^c^	45±5 ^c^	88±6 ^b^	130±3 ^c^
	Epp+FR	19±4 ^d^	36±2 ^d^	53±8 ^c^	95±0 ^d^
Application of speed breeding in GH (day/night - Normal: natural light - Epp: 20/4 h)	Normal GH	28± ^a^	51±0 ^a^	71±0 ^a^	128±0 ^a^
	Epp GH	21±0 ^b^	28±0 ^b^	41±0 ^b^	84±0 ^b^
	Epp+FR GH	7±0 ^c^	21± ^c^	28±0 ^c^	71±0 ^c^

*Means ± SD with the same letter in the stages of each experiment are not significantly different at the P = 0.05 level according to Duncan’s multiple range test.

**Days after germination in the FR light intensity experiment.

**Table 3:** Plant height and number of fruits of *Capsicum annuum* ‘MicroPep Red’ (‘MR’) from far-red (FR) light intensity experiment, pepper-specific speed breeding system, and application of speed breeding in greenhouse (GH).

Experiment	Treatment	Plant height (cm)	No. of fruit
FR light intensity experiment (day/night: 20/4 h)	W45	9.3±0.6 ^a^	–
	W45+LFR	9.2±0.9 ^a^	–
	W45+MFR	10.6±0.9 ^b^	–
	W45+HFR	16.4±1.0 ^c^	–
Pepper-specific speed breeding system (day/night - Ctl: 12/12 h - Epp: 20/4 h)	Ctl	8.6±0.5 ^a^	1.7±0.8 ^a^
	Ctl+FR	21.3±0.8 ^b^	2.8±1.2 ^a^
	Epp	9.5±0.5 ^c^	2.7±1.0 ^a^
	Epp+FR	21.7±1.1 ^b^	3.0±1.7 ^a^
Application of speed breeding in GH (day/night - Normal: natural light - Epp: 20/4 h)	Normal GH	2.9±0.2 ^a^	3±0 ^a^
	Epp GH	2.9±0.2 ^b^	3±0 ^a^
	Epp+FR GH	5.5±0.4 ^b^	27.8±3.7 ^b^

*Means ± SD with the same letter in the stages of each experiment are not significantly different at the P = 0.05 level according to Duncan’s multiple range test.

### 2.5 Application of a speed breeding system in the greenhouse

For applying the pepper-specific speed breeding system in the greenhouse, a system was devised in the greenhouse at Seoul National University Farm (Suwon, Republic of Korea). The ‘MR’ seeds were directly sown in the 72-hole seedling tray and grown in each treatment of the greenhouses during the fall season in Suwon, Republic of Korea. The basic cultivation conditions, such as pot size, amount of media, composition of the media, and water management, remained consistent with those described in the “Extending the photoperiod experiment.” However, approximately 85 days after transplantation in the greenhouse, an additional granular fertilizer (Inovatec, Jeongeup, Republic of Korea) was applied at a rate of approximately 5 pellets per pot. The fundamental cultivation conditions, including pot size, amount of media, composition of the media, and water management, remained consistent with those described in the “Extending the photoperiod experiment”. For the normal greenhouse (Normal GH) condition, the natural light and temperature in Suwon Farm were used. For the treatments of extended photoperiod in GH (Epp GH) and extended photoperiod with supplemented FR in GH (Epp+FR GH), the 20 h photoperiod and 4 h dark period were used (**Table 1**). Three plants for each treatment were analyzed. The light spectrum of LED (Bissol, Seoul, Korea) was 400-700 nm for Epp GH and 400-772 nm for Epp+FR GH (**Table 1**). The ratio of red to FR was adjusted to 4.8 in Epp GH and 3.8 in Epp+FR GH (**Table 1**).

### 2.6 Genomic DNA extraction

Total genomic DNA (gDNA) was extracted from three young leaves of plants using a modified cetyltrimethylammonium bromide (CTAB) method (Porebski et al., 1997). The extracted DNAs were quantified with a NanoDrop 1000 spectrophotometer (NanoDrop Technologies, Wilmington, DE, USA). The gDNA concentration was measured and diluted to 20 ng·μL^–1^.

### 2.7 GWAS and candidate gene identification

The GWAS accessions were genotyped using the genotyping-by-sequencing (GBS) method based on the *PstI*/*MseI* and *EcoRI*/*MseI* restriction enzymes as previously described (Lee et al., 2020). The digested DNA was ligated to adapters and the libraries were amplified using ‘TA’ primers (Lee et al., 2020). The libraries were pooled in five tubes, and the pooled libraries were sequenced using an Illumina HiSeq2000 sequencing system (Illumina, San Diego, CA, United States) at Macrogen (Seoul, South Korea). Filtered raw reads of GWAS population accessions were aligned to the *C*. *annuum* ‘Dempsey’ reference genome (Lee et al., 2022) using the Burrows-Wheeler Aligner (Hong et al., 2020). Alignment procedures yielded various types of variants, from which single nucleotide polymorphisms (SNPs) were exclusively extracted. These SNPs underwent a filtering process conditioned on parameters of a quality score (QUAL) < 30, a mapping quality (MQ) < 30.00, a Strand Odds Ratio (SOR) > 4.000, and a depth of coverage (DP) < 3 to ensure data reliability. A further filtering step followed, operating under a Minor Allele Frequency (MAF) cutoff of 0.05% and a call rate threshold of 50%. This filtering procedure resulted in the acquisition of 221,487 SNPs for utilization in the GWAS. Prior to conducting the GWAS, an exploratory principal component analysis (PCA) of the genotypic data earmarked for GWAS revealed that two principal components accounted for over 95% of the population variance. This insight dictated the analytical strategy for the subsequent GWAS, integrating the two principal components to consider population structure appropriately. The GWAS based on the compressed mixed linear model (CMLM) was conducted using the R Package Genomic Association with default settings (Wang and Zhang, 2021). The significance threshold was set after the Bonferroni multiple-test correction, setting the significant threshold level (Bonferroni, 1936). Candidate gene prediction for flowering time was performed based on the ‘Dempsey’ reference genome (Lee et al., 2022).

### 2.8 Sequence analysis of GWAS candidate genes

PCR was performed using 5 μL of 5x GXL buffer, 100 ng of template DNA, 10 pmol of each primer, 2 μL of 10mM dNTPs, and 0.5 μL of Taq polymerase (PrimeStar GXL; Takara, Shiga, Japan). Primers were designed to amplify all the coding sequences of candidate genes. The amplified products were resolved in 1% agarose gel (Lonza, Lockland, ME, USA) and gel eluted and purified using a PCR Clean-up Kit (Cosmogenetech, Seoul, Korea). The amplicons were sequenced at Macrogen (Macrogen, Seoul, Korea) and analyzed using SeqMan (Ver 5.00, DNASTAR Inc., Madison, WI, USA).

### 2.9 Expression analysis of GWAS candidate genes using quantitative real-time PCR

For gene expression analysis, the young leaves (1 cm) of ‘MR’ grown in the pepper-specific speed breeding system were sampled when the fourth and the sixth leaf were initiated. The leaves were frozen immediately in liquid nitrogen after sampling. Total RNA was extracted from the leaves using the MG Total RNA extraction kit (MGmed, Seoul, Republic of Korea). Total RNA (1μg) was used for cDNA synthesis by reverse transcription (RT) PCR using AccuPower RT PreMix (Bioneer, Daejeon, Republic of Korea). For qRT-PCR, three biological replicates were used for each sample. Based on the exon sequencing results of the ‘MR’, primers for qRT-PCR have been designed. The identified variations in the exons have the potential to explain the rapid flowering of the ‘MR’. The qRT-PCR was performed using the primers AP2_qRT (Borovsky et al., 2015), WOX4_qRT, FT_qRT, and GI_qRT. The qRT-PCR was performed in a 20 μL reaction volume containing 2 μL of 5x diluted cDNA, 2 μL of 10mM dNTPs, 2 μL of 10x reaction buffer, 0.5 μL of SYTO 9 (Thermo Fisher Scientific Korea, Seoul, Republic of Korea), 0.5 μL of 10 pmol primers, and 0.4 μL of R Taq (Takara Bio). A Rotor-Gene 6000 real-time PCR thermocycler (Corbett Research, Sydney, Australia) was used with the following PCR amplification conditions: 95°C for 5 min; 45 cycles of 95°C for 30 s, 58°C for 30 s, and 72°C for 30 s. The relative expression levels of candidate genes were normalized against *CaUBIQUITIN* (DQ975458.1) using the primer UBQ_qRT (Borovsky et al., 2015). The reliability and reproducibility were ensured with three independent replicates per plant. Statistical analysis was conducted by Tukey’s honest significant difference (HSD) test for pairwise comparisons and significant differences of means (Tukey, 1977) using R program.

## 3 Results

### 3.1 A photoperiod extended to 20 h was the best condition for accelerating the flowering time

**Figure 1:**
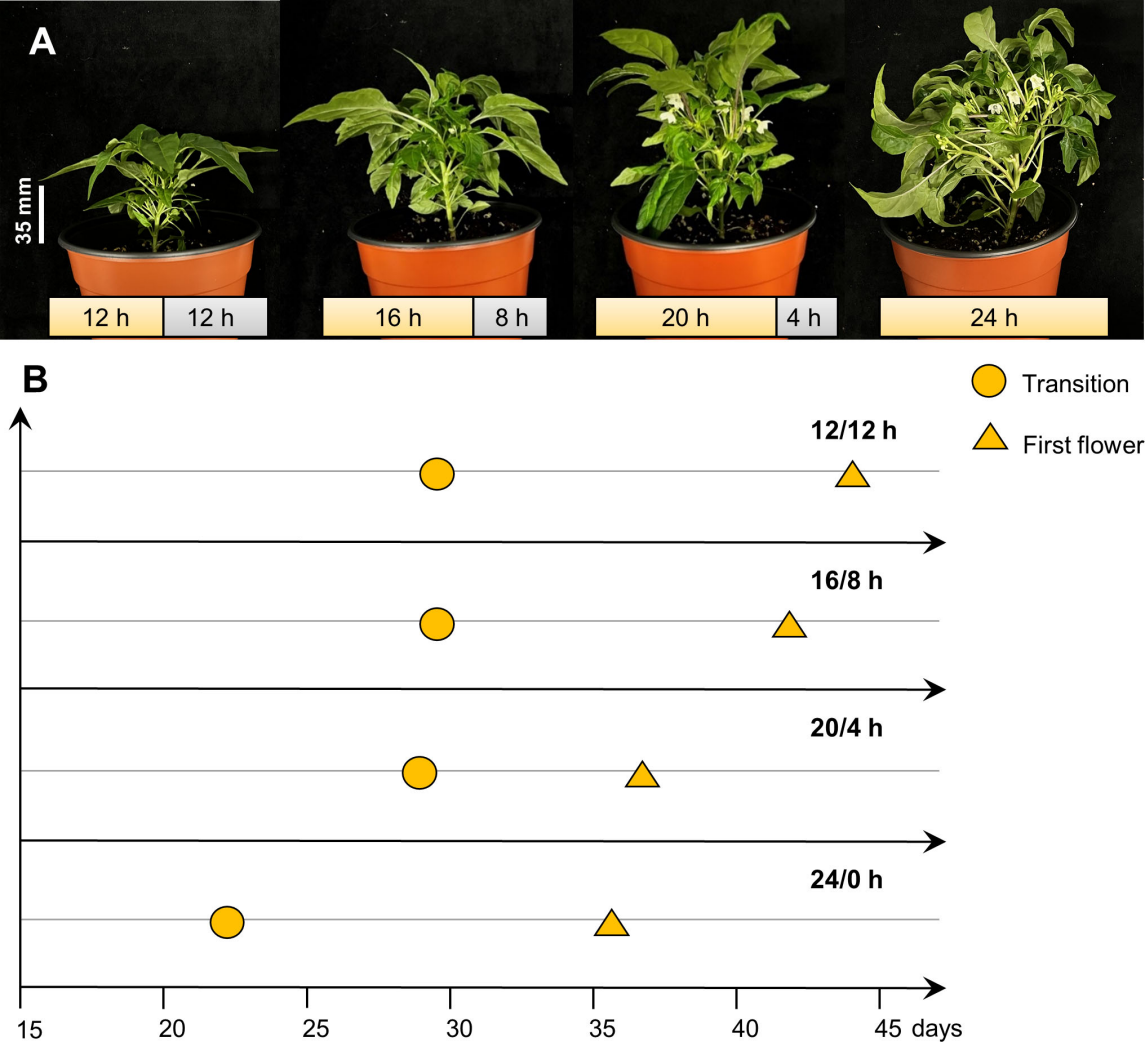
The growth and development of *Capsicum annuum* ‘MicroPep Red’ (‘MR’) in extending the photoperiod experiment. **(A)** ‘MR’ at 39 days after transplant (DAT) showed transition stage (appearance of flower buds) under the 12/12 h and 16/8 h of light/dark cycle. Concurrently, ‘MR’ showed flowering under the 20/4 h and 24/0 h of light/dark cycle. **(B)** Representative graph depicting the development stages of ‘MR’ under the various photoperiod conditions.

To find out the best photoperiod for the flowering of the pepper, as shown in **Figure 1**, pepper plants were grown under various conditions with 12 h, 16 h, 20 h, and 24 h of photoperiod. As the photoperiod extended, the ‘MR’ variety demonstrated a reduction in the number of days required for transition, indicating an accelerated emergence of floral meristem (**Table 2**). Flowering time was shortened by 6-7 days under the 20/4 h and 24/0 h conditions compared to the 12/12 h and 16/8 h conditions (**Table 2**). However, the plants under 24 h showed physiological disorders with wrinkled and yellowing leaves (**Supplementary Figure 3**). Thus, it was demonstrated that extending the day length to 20 h effectively promotes the flowering time and is the best photoperiod for speeding up the flowering of ‘MR’ (**Figure 1** and **Table 2**).

### 3.2 A lower R:FR ratio promoted the flowering of *Capsicum annuum*

**Figure 2:**
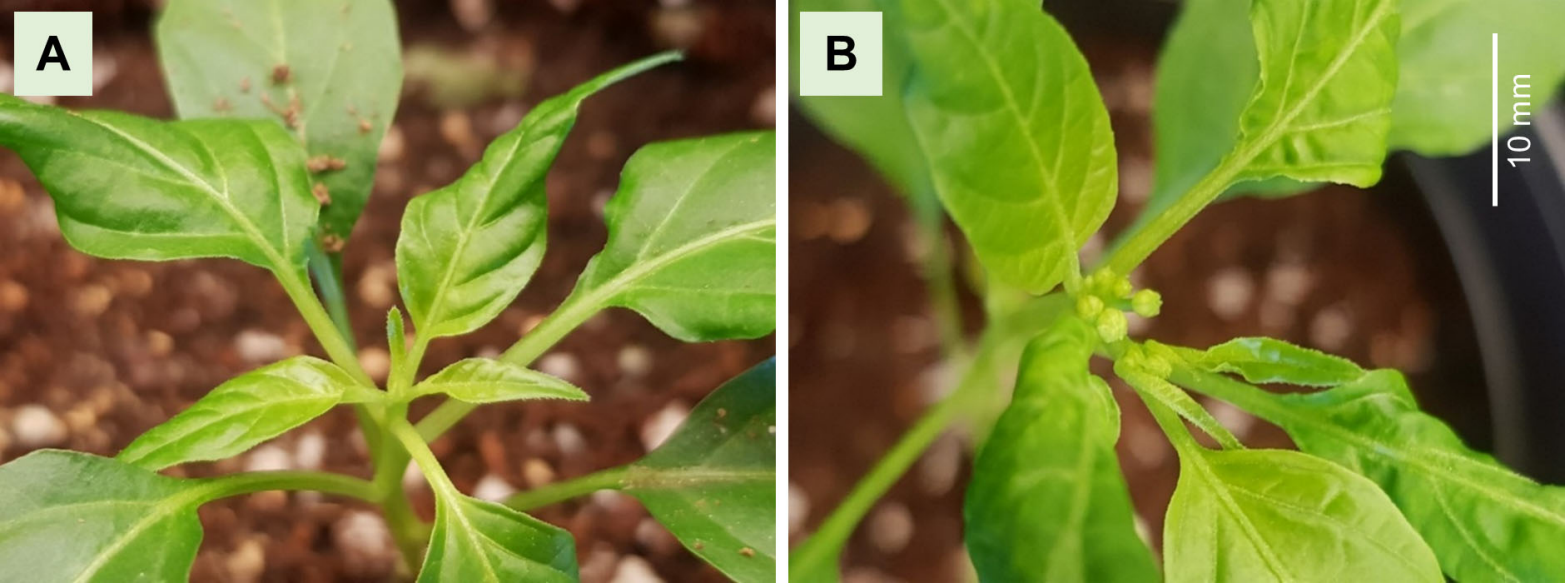
The appearance of flower buds in far-red (FR) light intensity experiment. **(A)** *Capsicum annuum* ‘MicroPep Red’ (‘MR’) at 23 days after germination showed vegetative shoot apical meristem under W45. **(B)** ‘MR’ at 23 days after germination showed inflorescence meristem under W45+HFR (R:FR = 0.3).

In the above experiment, it was found that extending the day length to 20 h could promote the flowering time of pepper. To further shorten the flowering time of ‘MR’, diverse R:FR ratios were tested. The plants under higher FR light treatment showed accelerated floral induction (**Figure 2** and **Table 2**). FR light stimulation facilitated floral differentiation, as evidenced by a reduction in the number of days required for the transition of the ‘MR’ variety (**Table 2**). However, it is important to note that FR light also induced undesired stem elongation. As the intensity of FR light increased, there was a significant promotion of stem elongation (**Table 3**). Overall, this study indicates that supplemented higher FR was effective for shortening the time to floral induction of ‘MR’, although unwanted stem elongation was observed.

### 3.3 The combination of extended photoperiod and FR light as a pepper-specific speed breeding system shortened the generation time effectively

**Figure 3:**
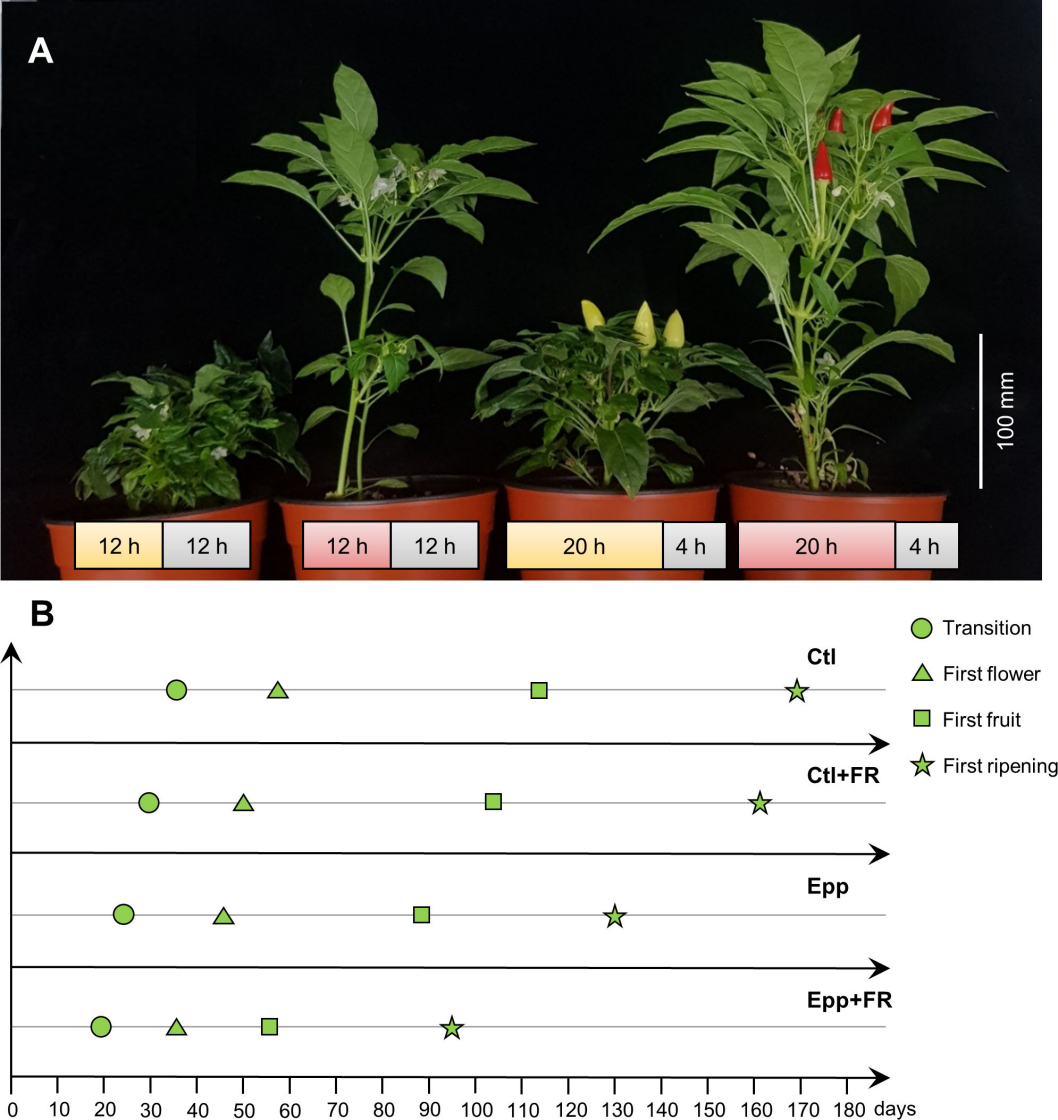
Growth and development of *Capsicum annuum* ‘MicroPep Red’ (‘MR’) in pepper-specific speed breeding system. **(A)** ‘MR’ at 100 days after transplant (DAT) under the Ctl (12/12 h and 26/20°C of light/dark cycle), Ctl+FR (12/12 h, 26/20°C of light/dark cycle, and supplemented far-red (FR) light), Epp (20/4 h and 26/20°C of light/dark cycle), Epp+FR (20/4 h, 26/20°C of light/dark cycle, and supplemented FR). **(B)** Representative graph depicting the development stages of ‘MR’ under the treatments (Ctl, Ctl+FR, Epp, and Epp+FR).

Through extending the photoperiod experiment and the FR light intensity test, it was found that increasing the light period to 20 h and higher FR light intensity could promote the flowering of ‘MR’. Therefore, to shorten pepper generation time dramatically, a pepper-specific speed breeding system was devised by combining extended photoperiod and supplementing FR light. A 20 h photoperiod and an R:FR ratio of 0.3 was adopted from the previous experiment, which was effective in promoting transition and flowering. Compared to other treatments (Ctl, Ctl+FR, Epp), transition, flowering, fruit, and ripening time were significantly reduced under Epp+FR treatment (**Figure 3** and **Table 2**). Specifically, compared to Ctl (56 ± 2 DAT), the combination of extended photoperiod and FR light (Epp+FR) shortened the flowering time significantly (36 ± 2 DAT) (**Figure 3** and **Table 2**). In addition to transition and flowering, fruit setting and ripening times were also significantly decreased in Epp+FR, suggesting that the generation time of pepper can be remarkably reduced due to a combination of extended photoperiod and supplemented FR, which can be a speed breeding system suitable for pepper (**Figure 3**). In addition, there were significant differences in stem length among the treatments (Ctl, Ctl+FR, Epp, and Epp+FR). Plants grown under FR light (Ctl+FR and Epp+FR) had higher stem lengths as compared to those grown under other conditions without FR light (non-FR) (**Figure 3** and **Table 3**).

### 3.4 Application of a pepper-specific speed breeding system in a greenhouse accelerated the breeding cycle of *Capsicum annuum*

**Figure 4:**
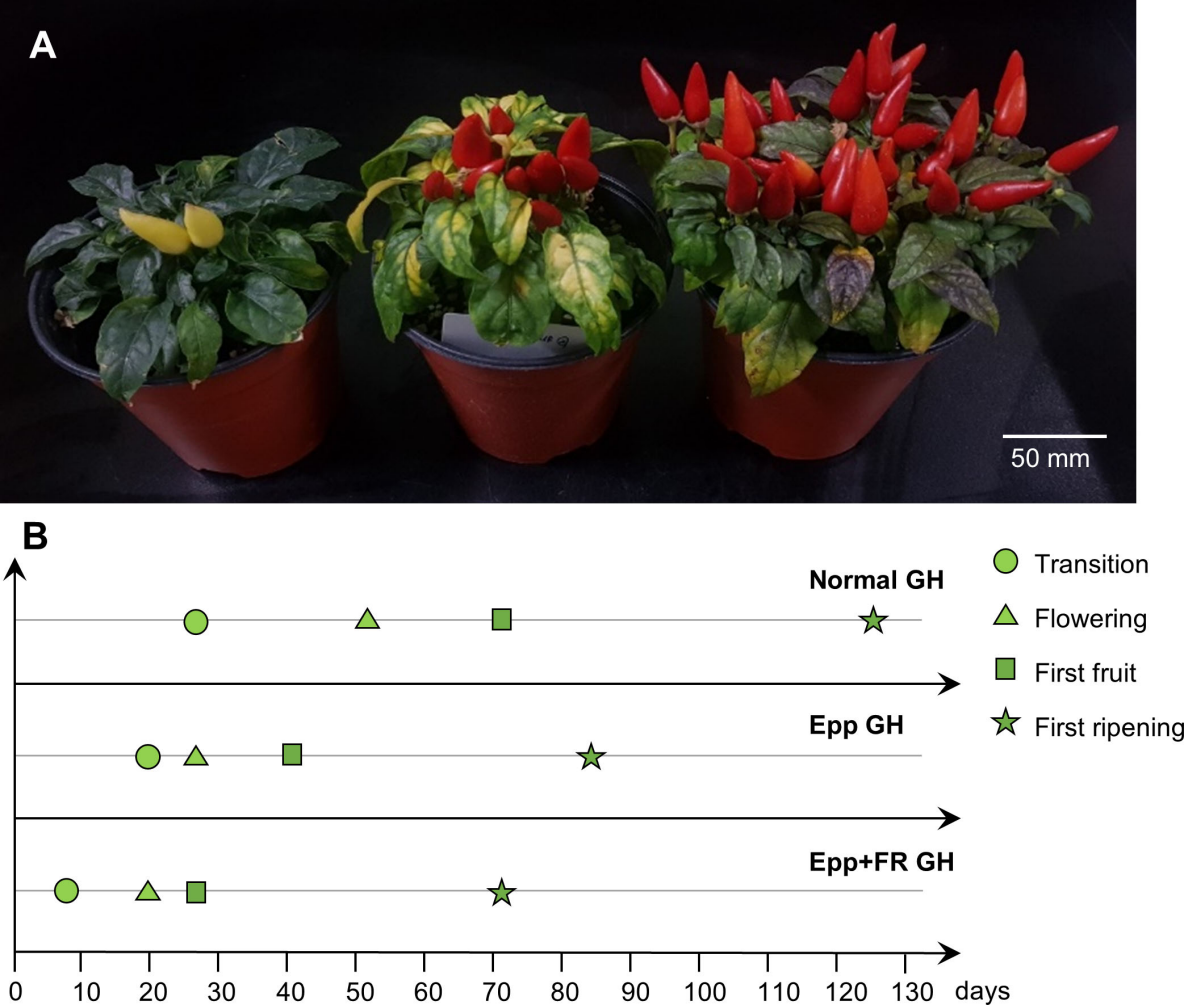
Growth and development of *Capsicum annuum* ‘MicroPep Red’ (‘MR’) from application of speed breeding in greenhouse (GH). **(A)** ‘MR’ at 99 days after transplant (DAT) under the Normal GH (Suwon farm condition), Epp GH (20/4 h of light/dark cycle), and Epp+FR GH (20/4 h of light/dark cycle and supplemented FR). **(B)** Representative graph depicting the development stages of ‘MR’ under the treatments (Normal GH, Epp GH, and Epp+FR GH).

Employing the results of the experiments in the chambers, speed breeding conditions with extended light and supplemented FR light were applied in the greenhouse. Since it was confirmed that the plant height was elongated when the FR light was higher than the red light in ‘MR’, an R:FR ratio of 3.8 was set in the greenhouse to prevent over-growth (Liu et al., 2022). Days to transition, flowering, first fruit, and first ripening were significantly shortened under Epp+FR GH compared to Normal GH and Epp GH (**Figure 4**). Days to flowering was 51, 28, and 21 DAT under Normal, Epp, and Epp+FR GH, respectively (**Table 2**). Days to first fruit and ripening were 28 and 71 DAT under Epp+FR GH (**Table 2**). Concurrently, the corresponding plants under Normal GH had only reached floral induction, while the plants under Epp+FR GH had already reached the first fruit stage (**Figure 4** and **Supplementary Figure 4**). Unlike the stem elongation under the higher R:FR of the FR light intensity experiment (**Figure 3** and **Table 3**), the ratio of R:FR at Epp+FR GH did not cause stem elongation (**Figure 4** and **Table 3**). In addition to speeding up the flowering, Epp+FR condition upregulated the number of fruits, which is important to harvest more seeds for breeding peppers (**Table 3**).

### 3.5 Genome-wide association study provided candidate flowering-time genes for *Capsicum annuum*

Through previous experiments, it was confirmed that pepper generation time is different depending on the day length and the presence or absence of FR. In particular, growth and development were promoted the most in the experimental group in which the photoperiod was prolonged and FR was supplemented. To demonstrate that different day lengths and light quality can be a genetic signal for the transition from vegetative to reproductive stage, as well as simply increasing photosynthesis, GWAS analysis was performed to determine the genetic loci associated with flowering time.

**Figure 5:**
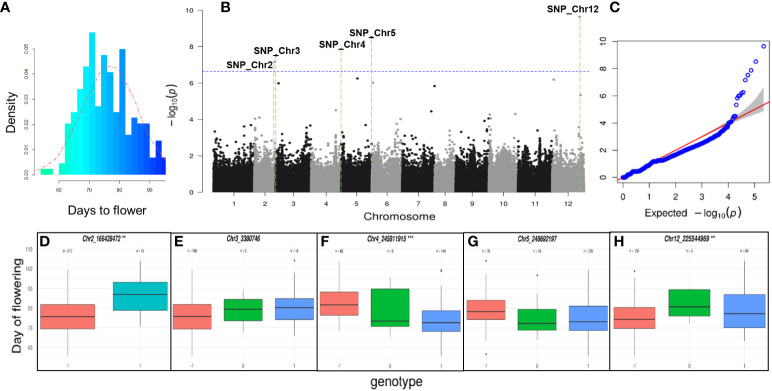
Genome-wide association analyses (GWAS) for flowering time. **(A)** Histogram for flowering time phenotype of pepper GWAS population. The histogram was drawn using the average flowering time of three-year data (2015, 2016, and 2017) in GWAS population. The red dotted line is the probability density curve following a normal distribution. **(B)** The 221,487 filtered single nucleotide polymorphisms (SNPs) detected from the 220 individuals of the GWAS population were used for detection of candidate genes. The GWAS were conducted using the R package Genomic Association with default settings. Blue line represents the threshold for GWAS significance after a Bonferroni correction. Five genome-wide significant SNPs (−log_10_ *P* > 7.158562) were identified on chromosomes 2, 3, 4, 5, and 12. **(C)** Quantile-quantile (QQ) plot of the data derived through FarmCPU model shown in the Manhattan plot. **(D–H)** The box plot showed the phenotypic variation of each SNP in the chromosomes 2, 3, 4, 5, and 12. The boxplot displays the phenotype variation in the GWAS population with the three different genotypes (-1: reference, 0: hetero, 1: alternative genotype) of 5 SNPs over the Bonferroni correction. Statistical significance was determined by ANOVA test (*P < 0.05, **P < 0.01, ***P < 0.001). The 3 SNPs in the chromosomes 2 (**), 4 (***), and 12 (**) showed significance in the phenotype variation among the 5 SNPs.

The phenotypic distribution of the flowering time using the GWAS population is illustrated in **Figure 5A**. This figure depicts the average flowering time over three years (2015, 2016, and 2017) for each C. annuum accession. Notably, the ‘MR’ accession exhibited an average flowering time of 68 ± 1.7 days under the normal greenhouse (traditional sunlight). The GWAS of the flowering-time traits was performed using a compressed MLM model (CMLM) as implemented in FarmCPU program with 221,487 high-quality SNPs and 220 C. *annuum* accessions. We identified 5 genome-wide significant SNPs (−log_10_ *P* > 7.158562). These SNPs were located on chromosomes 2, 3, 4, 5, and 12 (**Figures 5B, C**). The boxplots drawn with the genotypes of the 5 significant SNPs against the flowering-time phenotypes revealed that 3 SNPs on chromosomes 2, 4, and 12 were significantly associated with phenotype variation (**Figures 5D**-**H**). The gene annotation dataset was investigated to find significant flowering-related genes in the vicinity of the SNPs. The *APETALA2* (*AP2*), *WUSCHEL*-*RELATED HOMEOBOX4* (*WOX4*), *FLOWERING LOCUS T* (*FT*), and *GIGANTEA* (*GI*) genes were identified and selected as candidate genes for sequencing and expression analysis (**Supplementary Tables 3**–**5**).

### 3.6 Gene expression of *FT* and *GI* showed significant differences due to growth conditions in the pepper-specific speed breeding system

**Figure 6:**
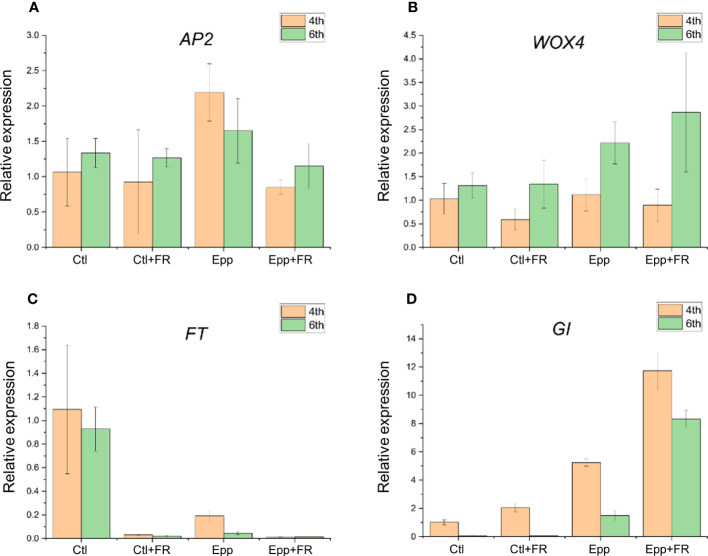
Quantitative RT-PCR results of candidate genes. Relative mRNA level of **(A)** *AP2*, **(B)** *WOX4*, **(C)** *FT*, and **(D)** *GI* in the Ctl, Ctl+FR, Epp, and Epp+FR at 4^th^ and 6^th^ leaves. Data for each group are means ± SE of three independent replicates. The *P*-values for significant differences between each treatment are specified in **Supplementary Tables 4, 5**.

Candidate gene expression patterns were analyzed in young leaves of ‘MR’ under Ctl and other treatments (Ctl+FR, Epp, and Epp+FR). The gene expression patterns of *AP2* and *WOX4*, which play an important role in floral development in Arabidopsis (Costanzo et al., 2014; Borovsky et al., 2015), did not show significant differences among environments (**Supplementary Tables 4, 5**). On the other hand, the expression levels of *FT* and *GI* were significantly different depending on growth condition (**Supplementary Tables 4, 5**). The *FT* gene found near the SNP on chromosome 5 had the highest expression in the Ctl (**Figure 6**). *GI* was highly expressed in Epp+FR, which extended photoperiod and supplemented FR light, and its expression level was the lowest under Ctl conditions (**Figure 6**). These gene expression results indicate that not only was flowering accelerated by increasing the amount of photosynthesis and photosynthetic efficiency due to extended photoperiod and adding FR (Zhen and van Iersel, 2017; Watson et al., 2018), but also the prolonged photoperiod or FR light can promote or inhibit the expression of flowering-related genes to change the flowering phenotype.

## 4 Discussion

Developing strategies for the rapid generation of homozygous lines is a dream for plant breeders. Watson et al. (2018) followed a new breeding approach, speed breeding, to shorten the generation time. In this approach, the authors exposed the plants to prolonged photoperiods, which resulted in a significantly shorter generation time for field crops such as wheat, barley, pea, and chickpea. However, this protocol is limited to long-day or day-neutral plants and does not consider other environmental controls such as light quality.

Extended daylight can clearly be beneficial for the flowering of some plant species and confirming the best day length is the key to success in speeding up the flowering. Extending the photoperiod, however, may also have some negative effects, because plants use the photoperiod as a signal for many other physiological processes. For example, tomato plants under continuous light showed leaf chlorosis and epinasty (Pham et al., 2019). These effects are largely on a species-specific basis. Continuous photosynthesis and accumulation of carbohydrates produced by continuous light may also result in negative feedback (Demers and Gosselin, 2000). These responses of plants to extended photoperiod and continuous light are very complex and are largely species or even cultivar specific (Velez-Ramirez et al., 2011). There is still much that is unknown about the mechanisms of plant responses to continuous light and the causes of the negative effects (foliar chlorosis, limited growth, productivity, leaf injury, etc.) (Sysoeva et al., 2010).

Recently, Jähne et al. (2020) proposed a protocol for short-day crops, such as soybean, rice, and amaranth, modulating optimal light conditions for each crop by adjusting blue, green, red, and FR light intensities. FR light did not affect the flowering time of soybean, but early flowering was observed under higher FR light in rice and amaranth. The flowering responses of plants can vary depending on environmental factors, such as various day-length and specific light spectrums and different plant species (Watson et al., 2018; Jähne et al., 2020); thus, species-specific breeding protocols need to be developed for each species, cultivar, and accession.

In this study, a speed breeding protocol for pepper was developed by extending the photoperiod to 20 h, controlling proper temperature, and supplementing FR light. Interestingly, this system demonstrated that prolonged photoperiod with FR lighting was the best condition for shortening the transition, flowering, fruit, and ripening time of pepper. This protocol shortened the flowering time to that of half of the plants grown in normal conditions. The time to first harvest was shortened by 75 DAT, which can shorten the breeding and research cycle of pepper remarkably. The effectiveness of the developed speed breeding system was observed not only in *C*. *annuum* ‘MicroPep Red’, the main variety used in this study but also in another species, *C*. *chinense* ‘Habanero’. **Supplementary Figure 5** illustrates that the flowering time was significantly promoted in the extended photoperiod+far-red light (Epp+FR) treatment compared to the control and extended photoperiod (Epp) treatments for ‘Habanero’. These findings imply that the speed breeding system developed in this study holds potential for application in other species within the *Capsicum* genus.

FR light was the key to this protocol and finding the best ratio of R:FR was essential. In the FR light intensity experiment, the lower R:FR ratio was effective for shortening flowering time in pepper, and the best R:FR ratio was 0.3. However, FR light can cause abnormal plant morphology of tall plants (Franklin, 2008). Plants with abnormally elongated stems are not suitable for growing in growth chambers and plant factories that have a limited height. Furthermore, breeders may face difficulties in growing and phenotyping plants due to lodging associated with elongated stems. To obtain an environment that can both promote flowering and maintain optimal stem growth in pepper, light quality and duration should be optimized. It is possible that the use of additional blue light along with Epp and FR light conditions (Epp+FR) could prevent the enhanced shoot growth due to blue light-mediated inhibition of hypocotyl growth regulated by cryptochromes (Franklin, 2008).

For the application of the developed speed breeding system in the greenhouse, we established an LED-controlled system. We set the photoperiod at 20 h and the R:FR ratio at 3.8 for accelerating the flowering time. We shortened the duration of seed to seed by 57 DAT with Epp+FR GH compared with Normal GH. When we applied this Epp+FR GH system in breeding the multiple disease resistant *C*. *annuum* and producing mature seeds of F_1_ plants, it took only 110 days and is predicted to be 880 days for developing BC_5_F_3_ lines.

The differences in plant responses to light can be explained by the expression of flowering-related genes. For example, in the case of Arabidopsis, FR light can promote *FT* expression (Cerdán and Chory, 2003; Halliday et al., 2003; Valverde et al., 2004) and flowering (Goto et al., 1991; Reed et al., 1993; Bagnall and King, 2001). These studies suggest that flowering-related genes can promote or inhibit flowering depending on the light conditions, therefore it is necessary to confirm gene expression under speed breeding, and relevant studies are lacking to date. To identify the genetic loci associated with flowering responses to day length and light and verify the gene expression patterns in the speed breeding system, GWAS and gene expression analysis were performed.

Multiple GWAS studies have elucidated the genetic structure related to key characteristics of pepper (*Capsicum* spp.), such as capsaicinoid content, fruit weight, and shape (Lozada et al., 2022a). Recently, Lozada et al. (2022b) performed a multi-locus GWAS analysis to investigate quantitative trait loci (QTL) associated with agronomic traits, including flowering time, in pepper (*Capsicum* spp.). Flowering time exhibited a strong correlation with the first fruit date trait, and shared QTL on chromosomes P1, P6, and P7 were identified. Relevant candidate genes were found to be involved in biological functions such as defense response, metabolic processes, oxidation-reduction, phosphorylation, and gene silencing (Lozada et al., 2022b). In this study, we identified five significant SNPs that were located in different positions compared to previous GWAS studies. Among them, *AP2* (located on chromosome 2), *WOX4* (located on chromosome 4), *FT* (located on chromosome 5), and *GI* (located on chromosome 12) were selected as candidate genes. Notably, *AP2* is the same locus as *CaAP2* introduced in a previous paper (Borovsky et al., 2015). As *CaAP2* acts as a flowering repressor in pepper, it was expected that *AP2* gene expression would be lowered in the Epp+FR treatment; however, *CaAP2* expression did not change significantly depending on the environment. A similar result was observed for the *CaWOX4* gene, a member of the *WOX* gene family. The *WOX4* protein contains the distinct WUS-box motif (van der Graaff et al., 2009), which is essential for shoot stem-cell population maintenance or differentiation, lateral organ formation, floral patterning, and embryogenesis (Kamiya et al., 2003; Haecker et al., 2004).

Contrary to *AP2* and *WOX4*, significant differences were found in the expression of *GI* and *FT* under different treatment conditions. Expression of *GI*, a flowering promoter in Arabidopsis (Mizoguchi et al., 2005), was significantly increased in the experimental conditions in which FR was applied with Epp. It was expected that *FT* gene expression would be enhanced in the FR condition since it is a representative flowering promoter and considered to be a florigen in Arabidopsis (Corbesier et al., 2007); however, expression of *CaFT* was inhibited in Epp+FR. This is because the functions of the flowering gene homologs in different plant species can be very different (Borovsky et al., 2015). Therefore, it can be inferred that the *FT* gene in *C*. *annuum* functions to delay flowering. To clarify the role of these candidate genes, further functional studies are required to better understand the molecular mechanisms by which these genes are involved in flower transition.

In conclusion, our results demonstrate the usefulness of the new speed breeding protocol developed for pepper. Pepper speed breeding can shorten the crop period to half that normally required for pepper, and thus shorten the crossing and inbreeding phase of breeding programs and enable breeders and scientists to save cost and time in their research programs. The shortened breeding cycle, made possible by speed breeding, leads to decreased resource requirements linked to extended cultivation periods, labor expenditure, and maintenance expenses. Furthermore, the shortened breeding time empowers breeders and scientists to rapidly assess and evaluate different genetic combinations, facilitating prompt decision making and optimal allocation of their research resources. In addition, breeding technologies such as marker-assisted or genomic selection can be incorporated into this speed breeding system to accelerate the development of pepper cultivars. The developed speed breeding protocol exhibits exceptional flexibility, making it highly adaptable for farm implementation. By incorporating this protocol into their farming operations, growers have the opportunity to innovate their cultivation techniques, leading to expedited yields and heightened overall efficiency. Furthermore, we located a new flowering gene locus for *C*. *annuum* and revealed the changes in gene expression during the speed breeding treatments. The significant associations identified herein provide a basis for further GWAS and mapping efforts to determine causal genetic variants and to clarify how the associated genes affect flowering transition in pepper.

## Data availability statement

The datasets presented in the study are deposited in the NABIC repository (https://nabic.rda.go.kr/nolog/NV-0799-000001/snpVcfView.do), accession number NV-0799.

## Author contributions

Conceived and designed the experiments: HC, SB, HL, B-CK. Performed the experiments: HC, SB, GK, KL, B-CK. Analyzed the data: HC, GK, B-CK. Wrote the paper: HC, JV, J-KK, B-CK.
