# Supplementary material for: Development of a speed breeding protocol with flowering gene investigation in pepper (Capsicum annuum)
Source: Front Plant Sci. 2023 Sep 18;14:1151765. doi: 10.3389/fpls.2023.1151765 (PMC10569693; doi:10.3389/fpls.2023.1151765)
Supplement: Supplementary file 1 [file Presentation_1.pptx]

## Slide 1
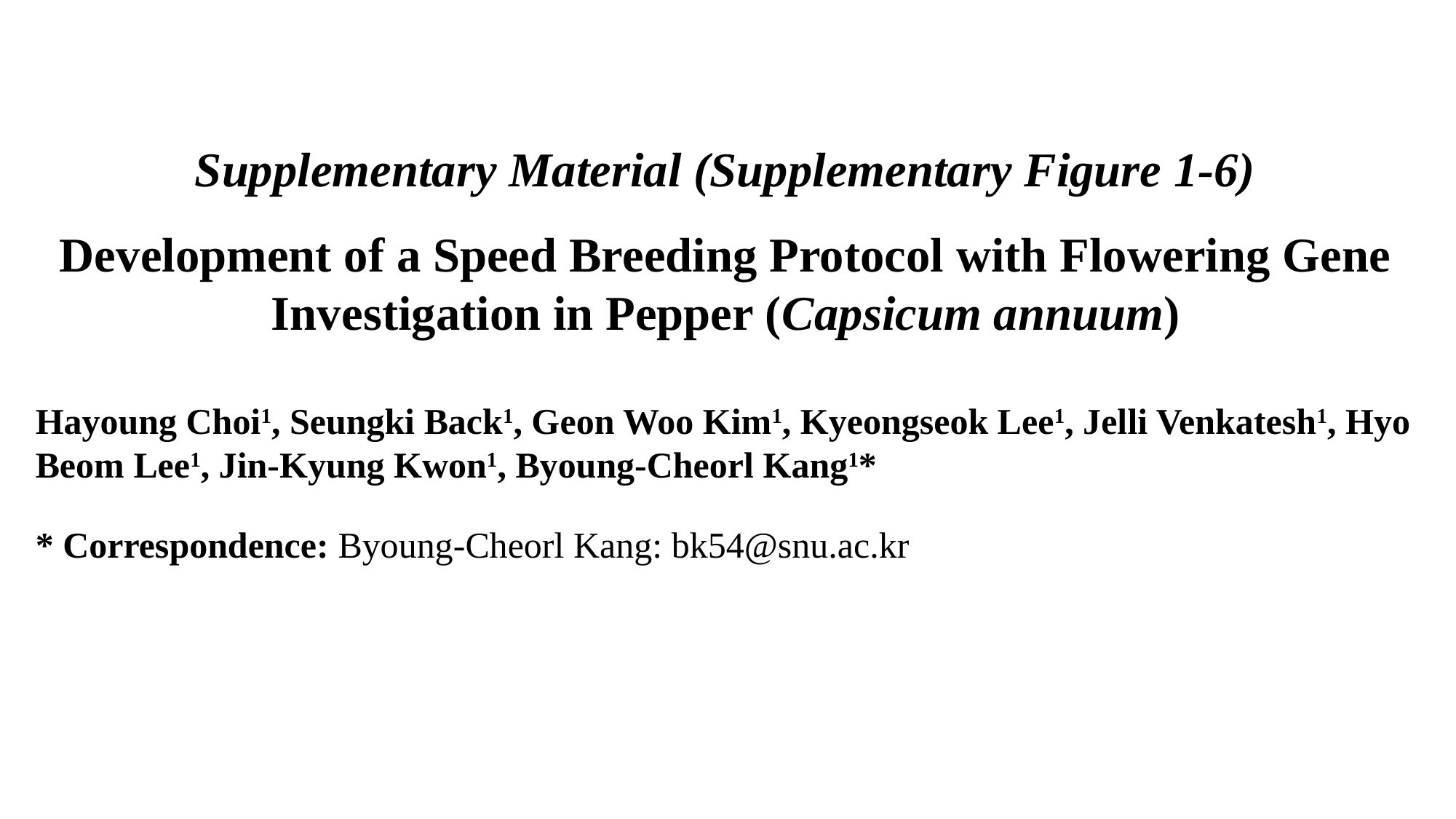

Supplementary Material (Supplementary Figure 1-6)
Development of a Speed Breeding Protocol with Flowering Gene Investigation in Pepper (Capsicum annuum)
Hayoung Choi1, Seungki Back1, Geon Woo Kim1, Kyeongseok Lee1, Jelli Venkatesh1, Hyo Beom Lee1, Jin-Kyung Kwon1, Byoung-Cheorl Kang1*
* Correspondence: Byoung-Cheorl Kang: bk54@snu.ac.kr

## Slide 2
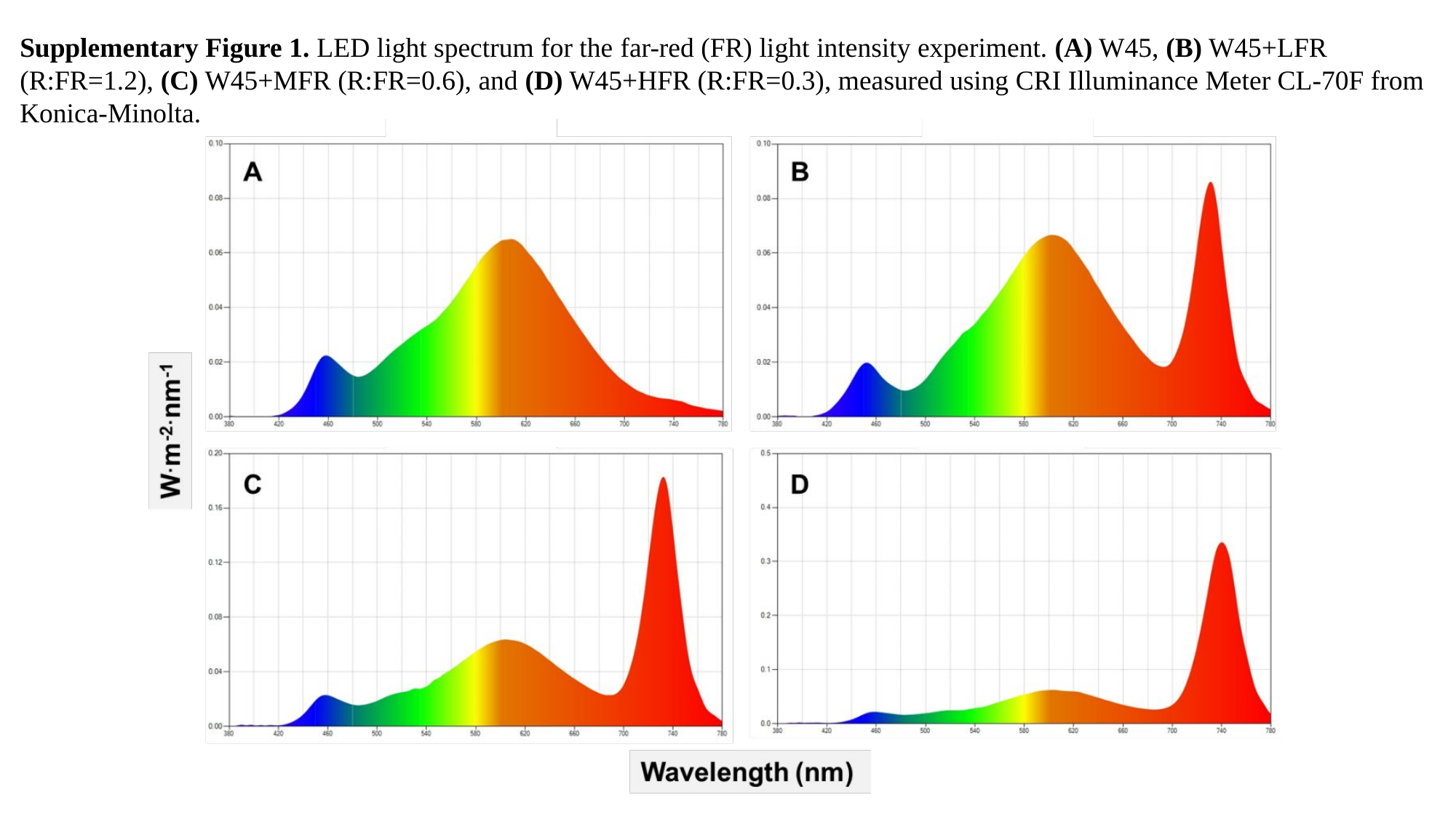

Supplementary Figure 1. LED light spectrum for the far-red (FR) light intensity experiment. (A) W45, (B) W45+LFR (R:FR=1.2), (C) W45+MFR (R:FR=0.6), and (D) W45+HFR (R:FR=0.3), measured using CRI Illuminance Meter CL-70F from Konica-Minolta.

## Slide 3
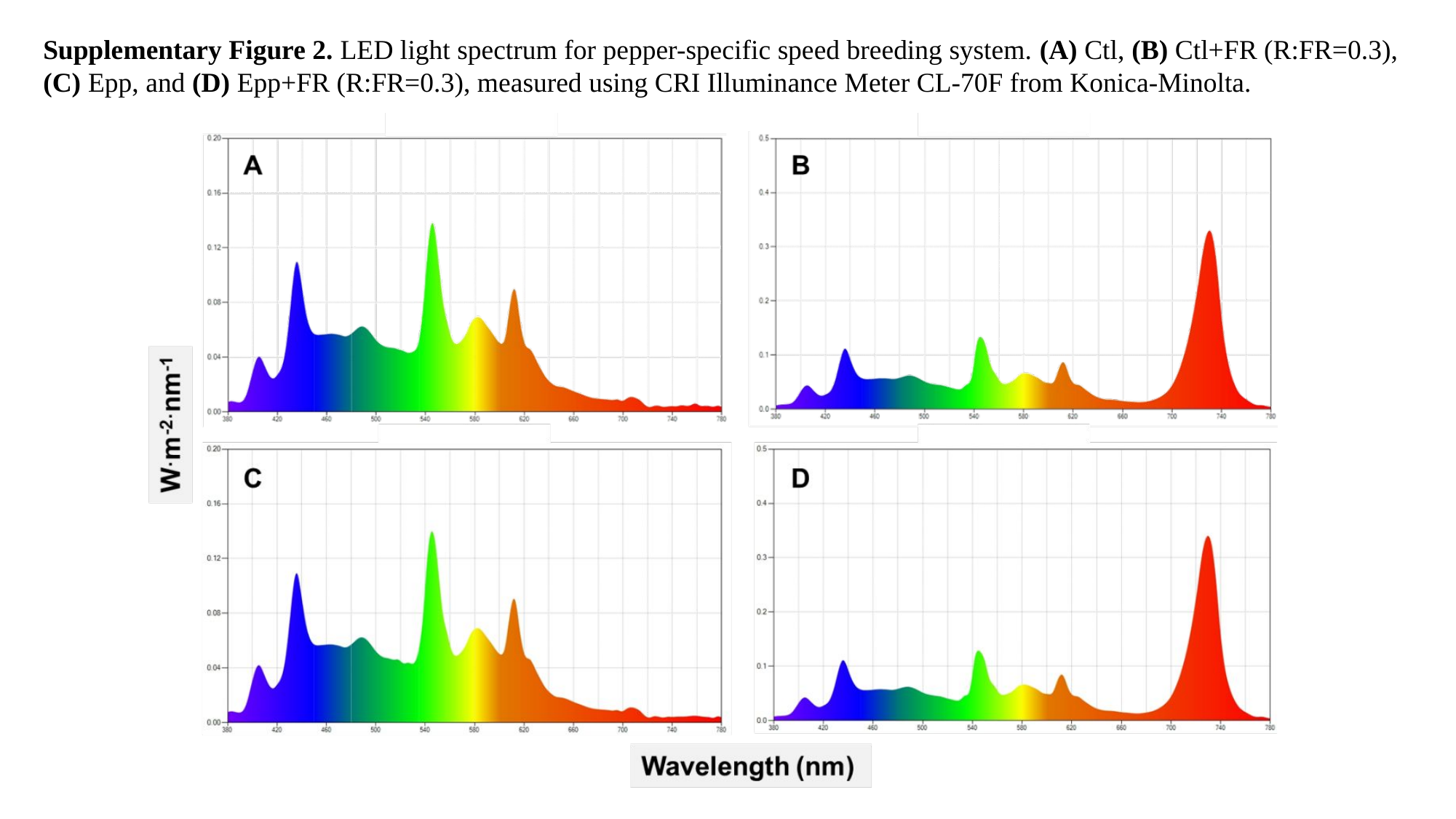

Supplementary Figure 2. LED light spectrum for pepper-specific speed breeding system. (A) Ctl, (B) Ctl+FR (R:FR=0.3), (C) Epp, and (D) Epp+FR (R:FR=0.3), measured using CRI Illuminance Meter CL-70F from Konica-Minolta.

## Slide 4
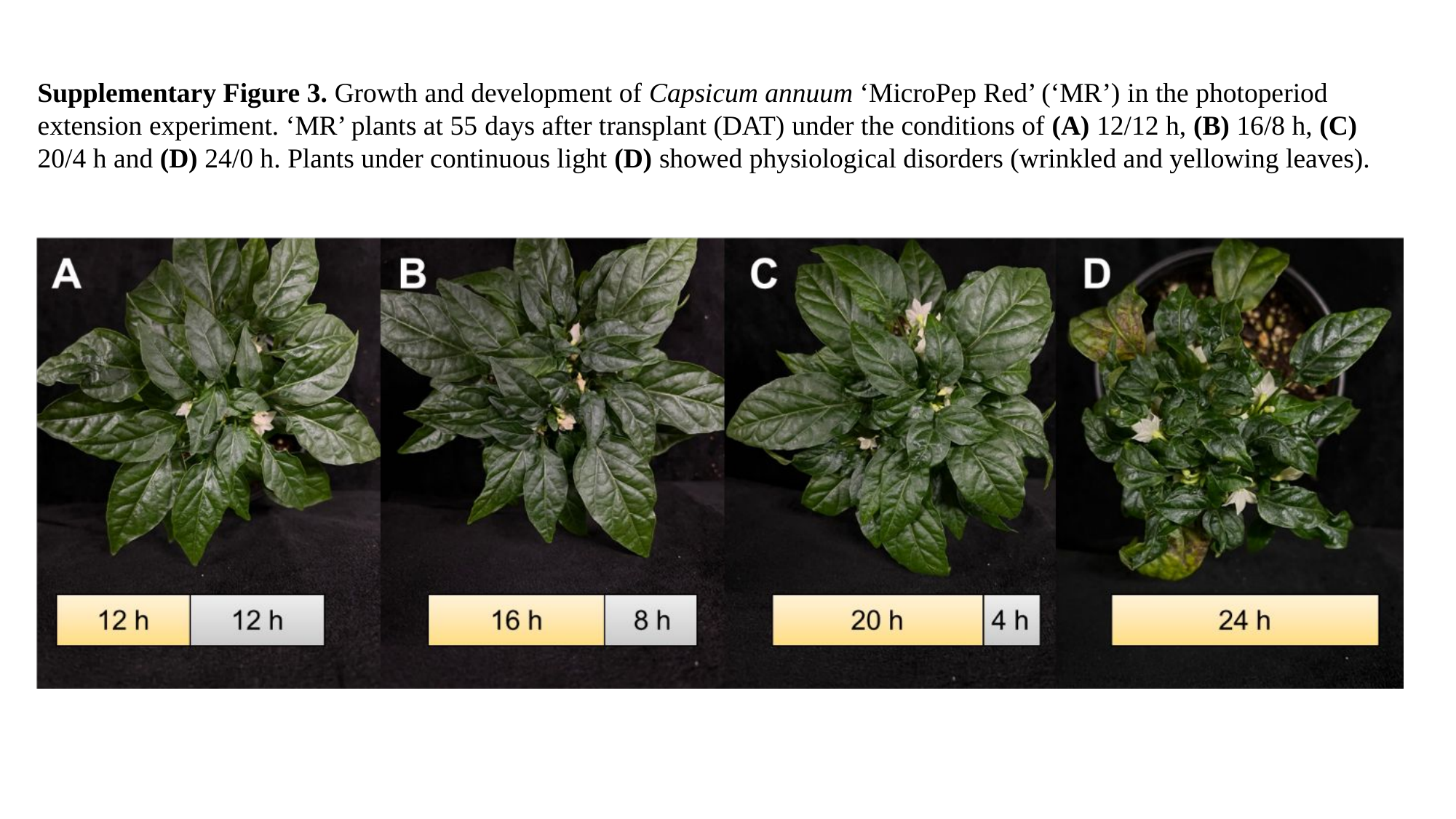

Supplementary Figure 3. Growth and development of Capsicum annuum ‘MicroPep Red’ (‘MR’) in the photoperiod extension experiment. ‘MR’ plants at 55 days after transplant (DAT) under the conditions of (A) 12/12 h, (B) 16/8 h, (C) 20/4 h and (D) 24/0 h. Plants under continuous light (D) showed physiological disorders (wrinkled and yellowing leaves).

## Slide 5
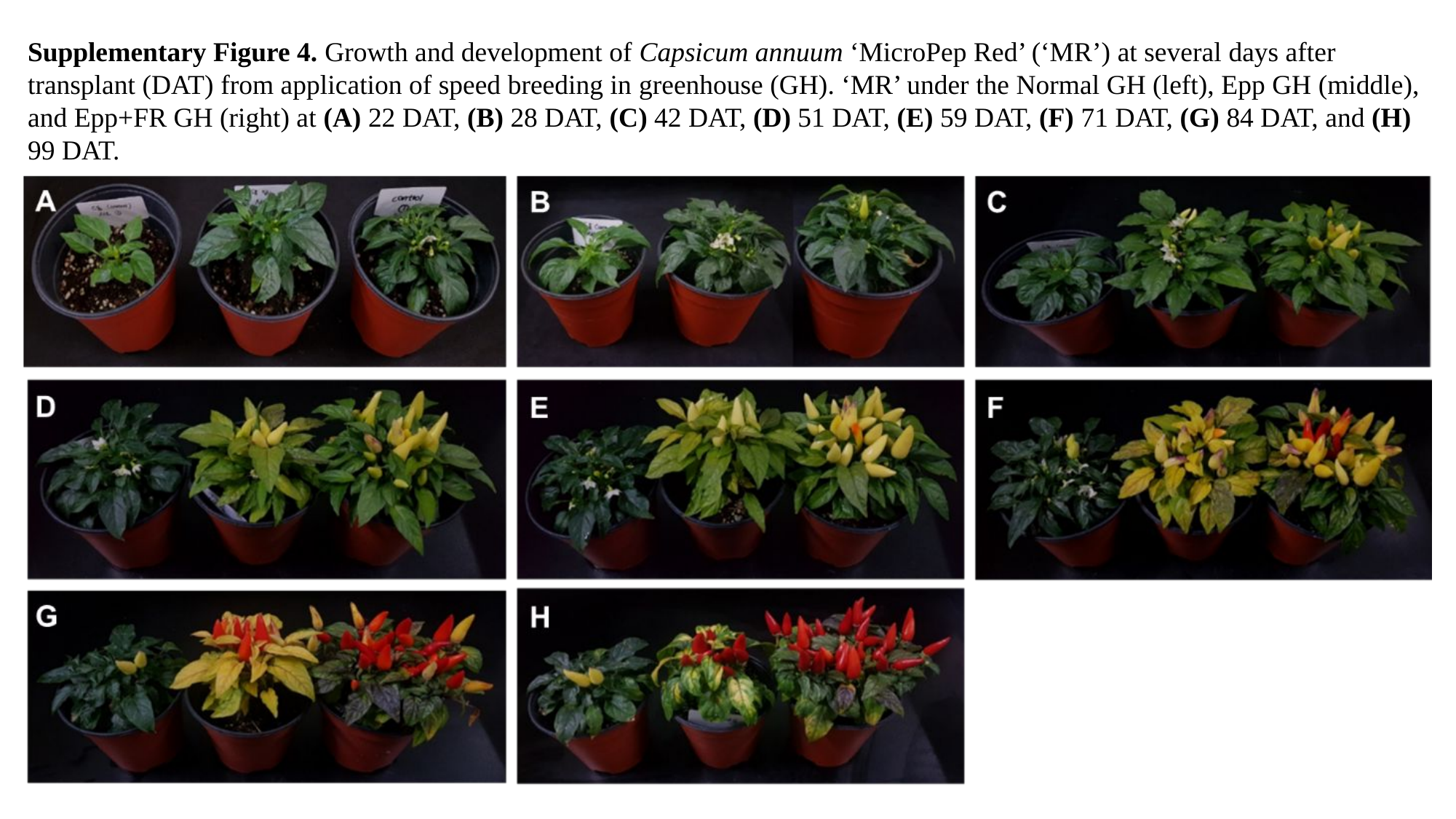

Supplementary Figure 4. Growth and development of Capsicum annuum ‘MicroPep Red’ (‘MR’) at several days after transplant (DAT) from application of speed breeding in greenhouse (GH). ‘MR’ under the Normal GH (left), Epp GH (middle), and Epp+FR GH (right) at (A) 22 DAT, (B) 28 DAT, (C) 42 DAT, (D) 51 DAT, (E) 59 DAT, (F) 71 DAT, (G) 84 DAT, and (H) 99 DAT.

## Slide 6
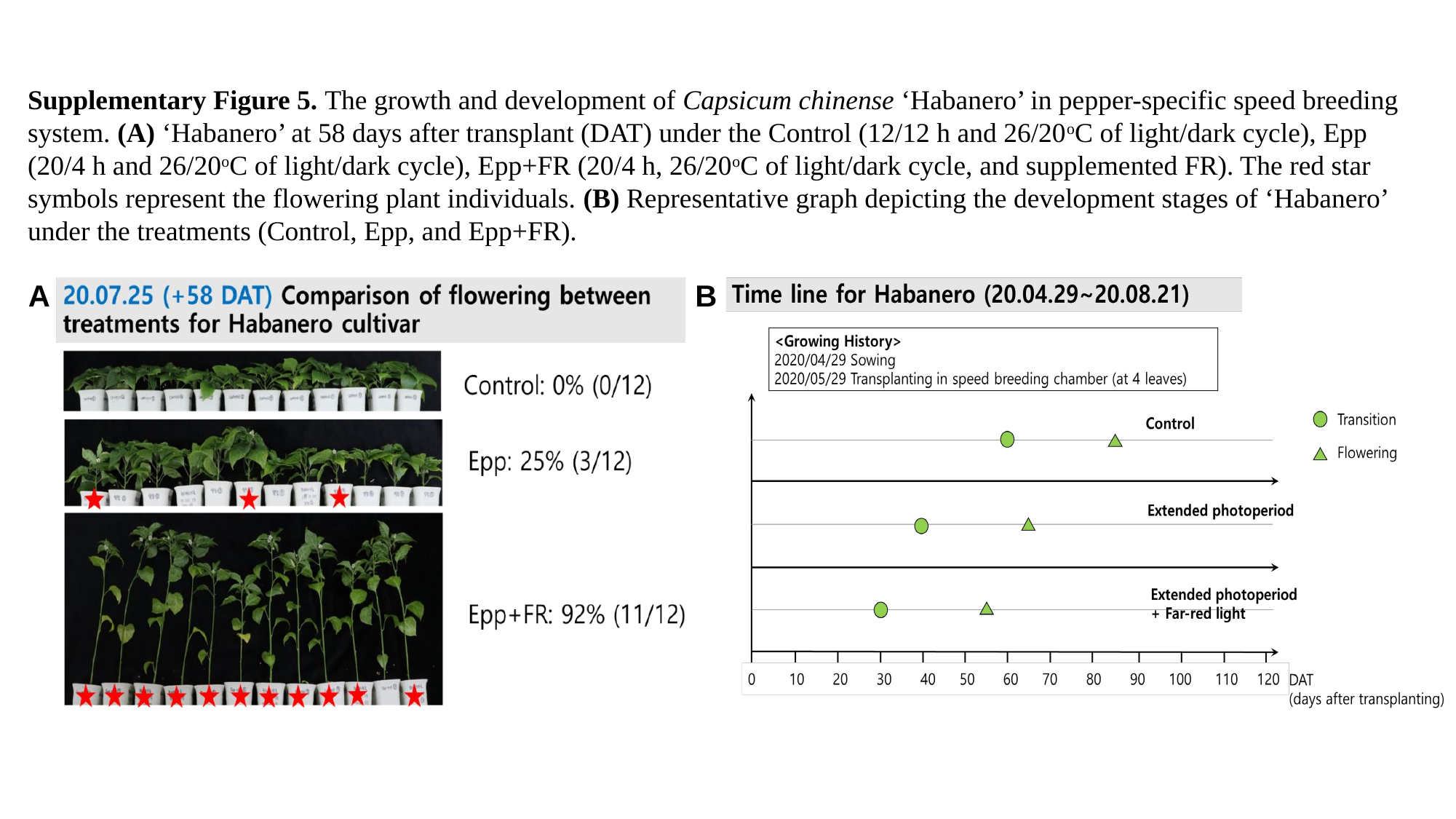

Supplementary Figure 5. The growth and development of Capsicum chinense ‘Habanero’ in pepper-specific speed breeding system. (A) ‘Habanero’ at 58 days after transplant (DAT) under the Control (12/12 h and 26/20oC of light/dark cycle), Epp (20/4 h and 26/20oC of light/dark cycle), Epp+FR (20/4 h, 26/20oC of light/dark cycle, and supplemented FR). The red star symbols represent the flowering plant individuals. (B) Representative graph depicting the development stages of ‘Habanero’ under the treatments (Control, Epp, and Epp+FR).
A
B

## Slide 7
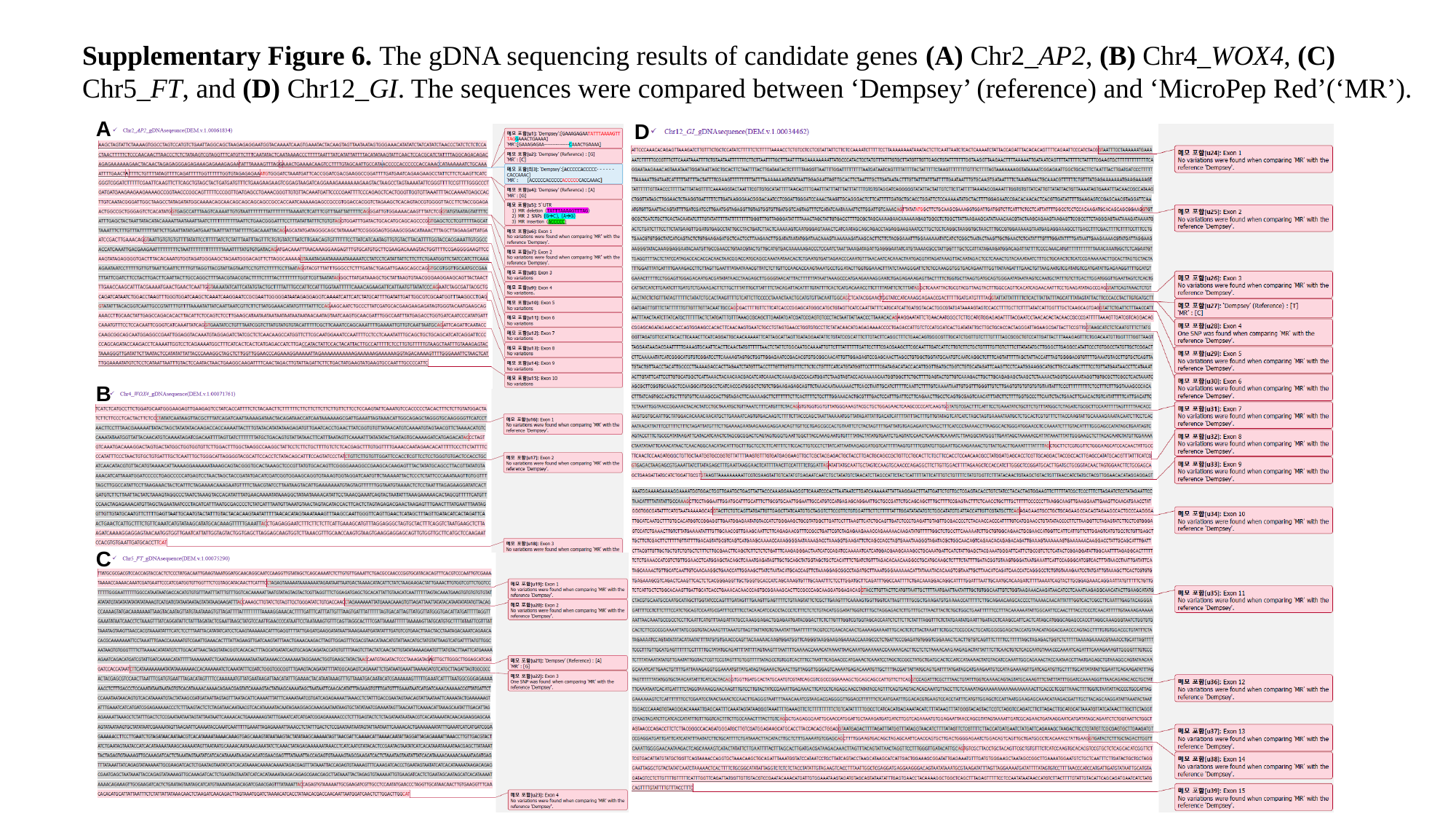

Supplementary Figure 6. The gDNA sequencing results of candidate genes (A) Chr2_AP2, (B) Chr4_WOX4, (C) Chr5_FT, and (D) Chr12_GI. The sequences were compared between ‘Dempsey’ (reference) and ‘MicroPep Red’(‘MR’).
A
D
B
C
