# Supplementary material for: Development of a speed breeding protocol with flowering gene investigation in pepper (Capsicum annuum)
Source: Front Plant Sci. 2023 Sep 18;14:1151765. doi: 10.3389/fpls.2023.1151765 (PMC10569693; doi:10.3389/fpls.2023.1151765)
Supplement: Supplementary file 2 [file Table_1.docx]

Supplementary Material

Development of a Speed Breeding Protocol with Flowering Gene Investigation in Pepper (*Capsicum annuum*)

Hayoung Choi^1^, Seungki Back^1^, Geon Woo Kim^1^, Kyeongseok Lee^1^, Jelli Venkatesh^1^, Hyo Beom Lee^1^, Jin-Kyung Kwon^1^, Byoung-Cheorl Kang^1^*

*** Correspondence:** Byoung-Cheorl Kang: bk54@snu.ac.kr

# Supplementary Tables (1-5)

**Supplementary Table 1.** Locations of SNPs and nearby candidate genes from GWAS.

|  | **Chr.** | **Position (bp)** | ***P*-value** | **-log_10_(*P*)** |
| --- | --- | --- | --- | --- |
| SNP_Chr2 | 2 | 166,428,472 | 6.941255e-08 | 7.158562 |
| Similar to *AP2*: Floral homeotic protein *APETALA 2* (*Arabidopsis thaliana*) | 2 | 166,686,987 – 166,690,378 |  |  |
| SNP_Chr3 | 3 | 3,380,746 | 3.010697e-08 | 7.521333 |
| SNP_Chr4 | 4 | 245,811,915 | 1.341343e-08 | 7.872460 |
| Similar to *WOX4*: *WUSCHEL-RELATED HOMEOBOX 4* (*Arabidopsis thaliana*) | 4 | 237,627,279 – 237,629,301 |  |  |
| SNP_Chr5 | 5 | 248,692,197 | 3.030186e-09 | 8.518531 |
| Similar to *FT*: Protein *FLOWERING LOCUS T* (*Arabidopsis thaliana*) | 5 | 249,368,383 – 249,372,154 |  |  |
| SNP_Chr12 | 12 | 225,544,969 | 2.271953e-10 | 9.643601 |
| Similar to *GI*: Protein *GIGANTEA* (*Arabidopsis thaliana*) | 12 | 211,321,849 – 211,334,911 |  |  |

**Supplementary Table 2.** List of primers used for sequencing candidate genes.

| Primer | Primer sequence (5′ to 3′) | Sequencing region |
| --- | --- | --- |
| AP2_e1-10_F | TGCAGTCATACCATTACCTTAGGAG | exon 1, 2, 3, 4, 5, 6, 7, 8, 9, 10 |
| AP2_e1-10_R | CAACTGACTTTCCGTCATTACTCA | exon 1, 2, 3, 4, 5, 6, 7, 8, 9, 10 |
| AP2_e1-4_F | CGGGTCGGATCTTTTTCGAATTC | exon 1, 2, 3, 4 |
| AP2_e1-4 _R | GTGAGCTCTGGGAAAATTCGG | exon 1, 2, 3, 4 |
| AP2_e5-8 _R | GATAAGGGCAGATTGCTTCTGG | exon 5, 6, 7, 8 |
| AP2_e2_F | AAAGTGGCCTAGTCCATGTCTG | exon 2 |
| AP2_e2_R | ACCACATTTGTGTAAAGTCACCC | exon 2 |
| AP2_e9-10_F | GCCACCATCAAATTGACGAAGA | exon 9, exon 10 |
| AP2_e9-10_R | CAACTGACTTTCCGTCATTACTCA | exon 9, exon 10 |
| WOX4_e1-2_F | GGGGTGTTTTAGCCTTAGGTCA | exon 1, exon 2 |
| WOX4_e1-2_R | GGAGCTTTAGTGCACCCGTA | exon 1, exon 2 |
| WOX4_e3_F | ACACTAGCGTTTTTCATGTTCCAA | exon 3 |
| WOX4_e3_R | TGTGTCTAAATCACGTTGTGCC | exon 3 |
| FT_e1-2_F | CACGCATCTAGGGCTTCACA | exon 1, 2 |
| FT_e1-2_R | TGGGGTTCAATTGGACATAGC | exon 1, 2 |
| FT_e3_F | CAGTGCAGACAGATACCATGTG | exon 3 |
| FT_e3_R | TAAGTTGGACGCTCGTAGTGG | exon 3 |
| FT_e4_F | AACCTCATCAATGTATACACTCCG | exon 4 |
| FT_e4_R | CCCTACATCAAACCCTAGCTAGC | exon 4 |
| GI_e1_F | ACCGGCGATAAATGAGAGACC | exon 1 |
| GI_e1_R | AATCCGAGGATTGTCCCGTTC | exon 1 |
| GI_e1Seq_R | GAAGTGCAGCCAATTCTCCG | exon 1 |
| GI_e2_F | TTTACAACCGCCATAAGCTGG | exon 2 |
| GI_e2_R | GCCACACGTTCACAGGAAAG | exon 2 |
| GI_e3-6_F | CTGGAGTTGGAGACAATGACGA | exon 3, 4, 5, 6 |
| GI_e3-6_R | AGACAACCAGATGCAAACGC | exon 3, 4, 5, 6 |
| GI_e7_F | CCATGGGCTCTGTCTGGAAG | exon 7 |
| GI_e7_R | TTACAGTGGCGCTCAGGAAC | exon 7 |
| GI_e8-9_F | ACATCAACTCTAGCGCGGAC | exon 8, 9 |
| GI_e8-9_R | CAGGTGTACTCGAGCAACAGA | exon 8, 9 |
| GI_e10_F | TGTCATATGTGAGAATCAATCTGC | exon 10 |
| GI_e10_R | CTCCGAGTATGAGAGACGGC | exon 10 |
| GI_e11_F | TGGCAGAACTGCGAACCAT | exon 11 |
| GI_e11_R | TCAAATTGAAGGCGAGTGACC | exon 11 |
| GI_e12_F | ACTGTCTCACCAATGTAAACCCA | exon 12 |
| GI_e12_R | AATAGGTGGACTTCACGTGTCC | exon 12 |
| GI_e13-14_F | TAAGACGAGGGGTTGGACCT | exon 13, 14 |
| GI_e13-14_R | ACGAAGAACCGATGTGCTGA | exon 13, 14 |
| GI_e15_F | CAGTCAGTTGCTGATGGCCT | exon 15 |
| GI_e15_R | ACCCTGCATTCAACGCAAAT | exon 15 |

**Supplementary Table 3.** List of primers for qRT-PCR of candidate genes.

| Gene | Primer sequence (5′ to 3′) | Reference |
| --- | --- | --- |
| *AP2* | F: TCAGATTCAATACCCAAGCGGC  R:GCTGCAAATATTTGAGGAGGAAAATTG | (Borovsky et al., 2015) |
| *WOX4* | F: TCTGGATGCAATGGGAAGAGT  R:AAGTAGTGAGGGAAGAAGATAGTCC | Present study |
| *FT* | F: GACGTCCACCAGTACCACTC  R:CTACGAGAAACCAACACCATCG | Present study |
| *GI* | F: AGTGTCCTTTTAACCCATCCATGA  R:AGCTGAGCCAGCCTTTTGTA | Present study |
| *UBQ* | F: GCACAAGCACAAGAAGGTTAAG  R:GCACCACACTCAGCATTAGGA | (Borovsky et al., 2015) |

**Supplementary Table 4.** *P*-value of candidate gene expression (*AP2*, *WOX4*, *FT*, and *GI*) from Ctl, Ctl+FR, Epp, and Epp+FR at 4^th^ leaves.

| Gene |  | Ctl | Ctl+FR | Epp | Epp+FR |
| --- | --- | --- | --- | --- | --- |
| *AP2* | Ctl | - |  |  |  |
|  | Ctl+FR | 0.9850683 | - |  |  |
|  | Epp | 0.0823667 | 0.0507296 | - |  |
|  | Epp+FR | 0.9459265 | 0.9969552 | 0.0383518 * | - |
| *WOX4* | Ctl | - |  |  |  |
|  | Ctl+FR | 0.3682357 | - |  |  |
|  | Epp | 0.9866972 | 0.2432207 | - |  |
|  | Epp+FR | 0.946509 | 0.6478007 | 0.8198187 | - |
| *FT* | Ctl | - |  |  |  |
|  | Ctl+FR | 0.0062699 ** | - |  |  |
|  | Epp | 0.0158327 * | 0.8881612 | - |  |
|  | Epp+FR | 0.0056404 ** | 0.9997604 | 0.8523336 | - |
| *GI* | Ctl | - |  |  |  |
|  | Ctl+FR | 0.3093826 | - |  |  |
|  | Epp | 0.0002845 *** | 0.0019345 ** | - |  |
|  | Epp+FR | 0.0000002 *** | 0.0000005 *** | 0.000012 *** | - |

*, ** and *** mean *P* < 0.05, *P* < 0.01 and *P* < 0.001, respectively.

**Supplementary Table 5.** *P*-value of candidate gene expression (*AP2*, *WOX4*, *FT*, and *GI*) from Ctl, Ctl+FR, Epp, and Epp+FR at 6^th^ leaves.

| Gene |  | Ctl | Ctl+FR | Epp | Epp+FR |
| --- | --- | --- | --- | --- | --- |
| *AP2* | Ctl | - |  |  |  |
|  | Ctl+FR | 0.9922232 | - |  |  |
|  | Epp | 0.6045065 | 0.4575471 | - |  |
|  | Epp+FR | 0.8801873 | 0.9654617 | 0.2621954 | - |
| *WOX4* | Ctl | - |  |  |  |
|  | Ctl+FR | 0.9999487 | - |  |  |
|  | Epp | 0.4689984 | 0.4952468 | - |  |
|  | Epp+FR | 0.1142311 | 0.1226499 | 0.7022612 | - |
| *FT* | Ctl | - |  |  |  |
|  | Ctl+FR | 0.0000104 *** | - |  |  |
|  | Epp | 0.0000125 *** | 0.9906028 | - |  |
|  | Epp+FR | 0.0000099 *** | 0.9998274 | 0.9816606 | - |
| *GI* | Ctl | - |  |  |  |
|  | Ctl+FR | 0.9999304 | - |  |  |
|  | Epp | 0.0042123 ** | 0.0045097 ** | - |  |
|  | Epp+FR | 0 *** | 0 *** | 0 *** | - |

*, ** and *** mean *P* < 0.05, *P* < 0.01 and *P* < 0.001, respectively.
